# Nivolumab in previously treated advanced gastric cancer (ATTRACTION-2): 3-year update and outcome of treatment beyond progression with nivolumab

**DOI:** 10.1007/s10120-021-01173-w

**Published:** 2021-03-20

**Authors:** Narikazu Boku, Taroh Satoh, Min-Hee Ryu, Yee Chao, Ken Kato, Hyun Cheol Chung, Jen-Shi Chen, Kei Muro, Won Ki Kang, Kun-Huei Yeh, Takaki Yoshikawa, Sang Cheul Oh, Li-Yuan Bai, Takao Tamura, Keun-Wook Lee, Yasuo Hamamoto, Jong Gwang Kim, Keisho Chin, Do-Youn Oh, Keiko Minashi, Jae Yong Cho, Masahiro Tsuda, Taihei Nishiyama, Li-Tzong Chen, Yoon-Koo Kang

**Affiliations:** 1 Division of Gastrointestinal Medical Oncology, National Cancer Center Hospital, 5-1-1, Tsukiji, Chuo-ku, Tokyo, 104-0045 Japan; 2 Frontier Science for Cancer and Chemotherapy, Osaka University Graduate School of Medicine, Suita, Japan; 3 Department of Oncology, University of Ulsan College of Medicine, Asan Medical Center, Seoul, South Korea; 4 Department of Oncology, Taipei Veterans General Hospital, Taipei, Taiwan; 5 Division of Medical Oncology, Yonsei Cancer Center, Song-Dang Institute for Cancer Research, Yonsei University College of Medicine, Yonsei University Health System, Seoul, South Korea; 6 Division of Hematology and Oncology, Department of Internal Medicine, Linkou Chang Gung Memorial Hospital, Chang Gung University, Taoyuan, Taiwan; 7 Department of Clinical Oncology, Aichi Cancer Center Hospital, Nagoya, Japan; 8 Division of Hematology-Oncology, Department of Medicine, Samsung Medical Center, Sungkyunkwan University School of Medicine , Seoul, South Korea; 9 Department of Medical Oncology, National Taiwan University Cancer Center, Taipei, Taiwan; 10 Cancer Research Center, National Taiwan University College of Medicine, Taipei, Taiwan; 11 Department of Gastrointestinal Surgery, Kanagawa Cancer Center, Yokohama, Japan; 12 Present Address: Department of Gastric Surgery, National Cancer Center Hospital, Tokyo, Japan; 13 Division of Hematology and Oncology, Department of Internal Medicine, College of Medicine, Korea University, Seoul, South Korea; 14 Division of Hematology and Oncology, Department of Internal Medicine, China Medical University Hospital, China Medical University, Taichung, Taiwan; 15 Department of Medical Oncology, Faculty of Medicine, Kindai University, Osakasayama, Japan; 16 Present Address: Department of Medical Oncology, Kindai University Nara Hospital, Ikoma, Japan; 17 Division of Hematology and Oncology, Department of Internal Medicine, Seoul National University Bundang Hospital, Seoul National University College of Medicine, Seongnam, South Korea; 18 Keio Cancer Center, Keio University School of Medicine, Tokyo, Japan; 19 Kyungpook National University School of Medicine, Daegu, South Korea; 20 Department of Gastroenterology, Cancer Institute Hospital of the Japanese Foundation for Cancer Research, Tokyo, Japan; 21 Department of Internal Medicine, Seoul National University Hospital, Cancer Research Institute, Seoul National University College of Medicine , Seoul, South Korea; 22 Clinical Trial Promotion Department, Chiba Cancer Center, Chiba, Japan; 23 Department of Medical Oncology, Gangnam Severance Hospital, Yonsei University College of Medicine , Seoul, South Korea; 24 Department of Gastroenterological Oncology, Hyogo Cancer Center, Akashi, Japan; 25 Medical Information, Medical Affairs, Ono Pharmaceutical Co., Ltd., Osaka, Japan; 26 National Institute of Cancer Research, National Health Research Institutes, Tainan, Taiwan; 27 National Cheng Kung University Hospital, National Cheng Kung University, Tainan, Taiwan; 28 Department of Internal Medicine, Kaohsiung Medical University Hospital, Kaohsiung Medical University, Kaohsiung, Taiwan

**Keywords:** ATTRACTION-2, Gastric or gastroesophageal junction cancer, Nivolumab, Long-term efficacy, Treatment beyond progression

## Abstract

**Background:**

ATTRACTION-2 demonstrated that nivolumab improved overall survival (OS) vs placebo in patients with advanced gastric cancer treated with ≥ 2 chemotherapy regimens. However, its long-term efficacy and outcome of treatment beyond progression (TBP) with nivolumab have not been clarified.

**Methods:**

The 3-year follow-up data were collected. A subset analysis was performed to explore the efficacy of TBP by assessing postprogression survival (PPS) after the first event of disease progression.

**Results:**

Overall, 493 patients were randomized (2:1) to receive nivolumab (*n* = 330) or placebo (*n* = 163). With a median follow-up of 38.5 (range 36.1–47.5) months, OS of the nivolumab group was significantly longer compared to the placebo group (median 5.3 vs 4.1 months; 3-year survival rate, 5.6% vs 1.9%; hazard ratio [HR], 0.62 [95% confidence interval (CI) 0.50–0.75], *P* < 0.0001). The median OS of responders (*n* = 32) who achieved complete response or partial response was 26.7 months and the 3-year survival rate was 35.5% in the nivolumab group. Overall, 109 patients in the nivolumab group and 37 patients in the placebo group received TBP. PPS tended to be longer in the nivolumab group vs placebo group (median 5.8 vs 4.5 months; HR [95% CI], 0.69 [0.47–1.01], *P* = 0.057). In contrast, PPS was similar between both treatment groups in non-TBP patients (median 2.3 vs 2.2 months; HR 0.90, *P* = 0.42).

**Conclusions:**

Long-term efficacy of nivolumab was confirmed at the 3-year follow-up, and a survival benefit of TBP with nivolumab was suggested. Biomarkers for selecting patients suitable for TBP with nivolumab should be identified in the future.

**Supplementary information:**

The online version contains supplementary material available at 10.1007/s10120-021-01173-w.

## Introduction

Immune checkpoint inhibitors (ICIs) are a new option for anticancer treatment [1]. The efficacy of ICIs was evaluated initially in patients with previously treated [2] and then in untreated patients with metastatic melanoma without BRAF mutations [3], demonstrating improved overall survival (OS) in both populations. Subsequently, ICIs proved to be efficacious in several treatment lines for various cancer types, including lung cancer, renal cell carcinoma, and head and neck cancer [4–8].

ATTRACTION-2 was the first phase 3 study that demonstrated the efficacy and safety of nivolumab, a monoclonal antibody that blocks programmed death-1 (PD-1), as a third- or later-line treatment for patients with advanced gastric/gastroesophageal junction (G/GEJ) cancer compared with placebo [9]. In contrast, the JAVELIN Gastric 300 study of avelumab, a monoclonal antibody against programmed death-ligand 1 (PD-L1), failed to show superiority in OS compared with treatment of the physician’s choice [10]. While the two phase 3 trials of pembrolizumab showed marginally negative results in the first- and second-line setting for advanced G/GEJ cancer [11, 12], one of the two phase 3 trials of nivolumab showed an OS benefit of nivolumab in combination with standard chemotherapy versus chemotherapy alone in the first-line setting [13, 14]. It is speculated that the utility of ICIs may depend on the clinical setting in relation to the cancer types, treatment lines, and combination therapy, and treatment strategy with ICIs should be optimized in each situation.

One of the distinctive characteristics of ICIs is their long-term efficacy, which was reported in melanoma and lung cancer [8, 15–18]. While there are no reports on the long-term efficacy of salvage-line chemotherapy with cytotoxic agents such as irinotecan and trifluridine–tipiracil (TAS-102) in patients with advanced G/GEJ cancer, previous reports have demonstrated the efficacy and safety of ICIs at the 2-year follow-up with nivolumab in the ATTRACTION-2 study [19] and with pembrolizumab in the phase 2 KEYNOTE-059 study [20]. However, to date, no studies have reported the long-term efficacy of ICIs for advanced G/GEJ cancer. Another distinctive feature of ICIs is a phenomenon called pseudoprogression [21–25]. Treatment with ICIs activates lymphocytes, which may accumulate in the tumor, resulting in an apparent enlargement in the tumor size. Pseudoprogression is often difficult to distinguish from true tumor progression [26]. In this context, continuous treatment beyond progression (TBP) may be important until true disease progression. Furthermore, it has been reported that some patients with melanoma, renal cell carcinoma, and lung cancer may receive clinical benefits from TBP with nivolumab [27–29]. Indeed, in the ATTRACTION-2 study, after the first event of progressive disease (PD), TBP with nivolumab or placebo was permitted at the investigator’s expectation of clinical benefit and the patient’s consent for continuing the protocol treatment beyond the first evidence of PD [19]. Thus, TBP might have influenced the overall results of the ATTRACTION-2 study. The decision of selecting the next treatment at the first event of PD with nivolumab is clinically important, either TBP or a switch to other pharmacotherapies, in the salvage-line treatment of patients with advanced G/GEJ cancer. However, it is unclear whether TBP with nivolumab might have a survival benefit over placebo.

Here, we report the 3-year follow-up data of the ATTRACTION-2 study and a subset analysis of postprogression survival (PPS) after the first event of PD in all patients who received TBP in the nivolumab and placebo groups and according to the best overall response (BOR) and PD patterns.

## Methods

### Study design

ATTRACTION-2 was a randomized, double-blind, placebo-controlled, phase 3 study conducted at 49 sites in Japan, South Korea, and Taiwan (NCT02267343). The detailed procedure of the ATTRACTION-2 study has been published [9]. Briefly, eligible patients, who were aged ≥ 20 years, having an Eastern Cooperative Oncology Group (ECOG) performance status (PS) of 0 or 1, and unresectable advanced or recurrent G/GEJ cancer histologically confirmed to be adenocarcinoma refractory to or intolerant of ≥ 2 lines of standard chemotherapy, were randomized (2:1) to receive nivolumab or placebo [9].

Written informed consent was provided by all patients before enrollment and before TBP. The study was conducted in accordance with the Declaration of Helsinki and the Good Clinical Practice guidelines developed by the International Council for Harmonisation of Technical Requirements for Pharmaceuticals for Human Use [9].

### Procedures

The study treatment (intravenous infusion of nivolumab [3 mg/kg] or placebo every 2 weeks for 6 weeks [one cycle]) was continued until disease progression or the onset of toxicities requiring permanent treatment discontinuation. TBP was allowed for patients who met the following criteria at the first event of PD: expectation of clinical benefit, no rapid disease progression, tolerance to the study drug and preserved PS, no risk of interference from any intervention required to prevent serious complications due to disease progression with TBP, and provision of written informed consent for TBP [9].

Response was evaluated according to the Response Evaluation Criteria in Solid Tumors (RECIST) guidelines version 1.1 [30] using computed tomography (CT) or magnetic resonance imaging (MRI) every cycle for the first 10 cycles and every two cycles thereafter until permanent discontinuation of the study treatment due to any cause.

### Analysis

The primary endpoint was OS. Secondary endpoints were progression-free survival (PFS), BOR (complete response [CR], partial response [PR], stable disease [SD], and PD), objective response rate (ORR; the proportion of patients with confirmed CR or PR), and duration of response (DOR). Additionally, a subanalysis of OS by BOR was conducted in this study. Treatment-related adverse events (TRAEs) of special interest were also evaluated. Biomarkers including PD-L1 expression on tumor cells, tumor mutation burden (TMB), and microsatellite instability (MSI) were evaluated retrospectively.

PPS was calculated from the first event of PD to death from any cause, which was evaluated in all patients who received TBP and then in subpopulations according to the BOR and patterns of PD. The patterns of PD were classified as (1) increase in tumor size of existing target lesions by ≥ 20% without new lesions, (2) increase in tumor size by < 20% with new lesions, and (3) increase in tumor size by ≥ 20% with new lesions. Duration of TBP was defined as the interval between the first event of PD and the last administration of nivolumab or placebo. Additionally, PPS was also analyzed in patients not receiving TBP. Although there is no consensus for the definition of pseudoprogression with immune-oncology therapy, the change in tumor size after the first event of PD was assessed in all patients receiving TBP who had measurable lesions.

### Statistics

The Kaplan–Meier (KM) method was used to estimate OS, PFS, and PPS, which were compared between the two treatment groups using the stratified log-rank test with a one-sided significance level of 0.025. Hazard ratio (HR; 95% confidence interval [CI]) was calculated using the stratified Cox proportional hazards model. A subgroup analysis of OS by PD-L1, TMB, and MSI status was performed with an unstratified Cox model, including HR and corresponding 95% CIs, to examine the effect of treatment on OS. A spider plot was presented to evaluate the change in tumor size compared to the first event of PD among patients with measurable lesions and imaging data during TBP. All analyses were performed using SAS versions 9.3 and 9.4 (SAS Institute, Inc., Cary, NC, USA).

## Results

### Patient disposition

Overall, 601 patients were enrolled, of whom 493 (nivolumab 330, placebo 163) were randomized between November 4, 2014, and February 26, 2016. Baseline characteristics were presented in the previous publication [9]. The data cutoff date for this 3-year follow-up was February 17, 2019, with a median (range) follow-up period of 38.5 (36.1–47.5) months in survivors. The response was assessed in 399 patients having measurable lesions (nivolumab 268, placebo 131). Among the 493 patients, the proportion of patients who received post-study treatment after permanent discontinuation of study treatment increased at the 3-year follow-up than in the previous report; 54.2% (179/330) of patients in the nivolumab group and 47.2% (77/163) in the placebo group (pharmacotherapy, 42.1% [139/330] and 35% [57/163]; surgery, 20.9% [69/330] and 17.2% [28/163]; radiotherapy, 8.8% [29/330] and 10.4% [17/163], respectively).

### Efficacy

**Fig. 1:**
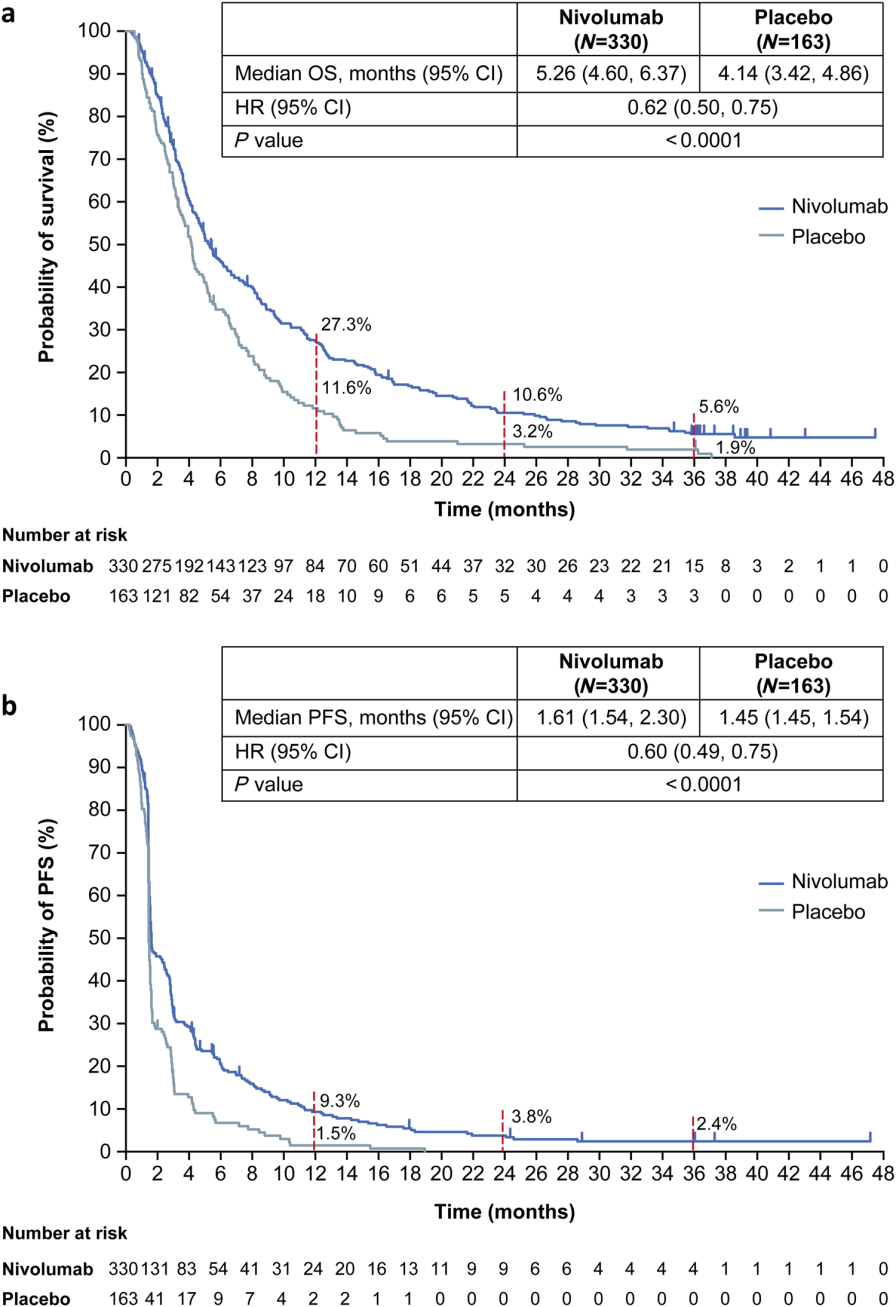
OS (**a**) and PFS (**b**) after 3 years of follow-up. Vertical marks on the curve indicate patients who were censored. *CI* confidence interval, *HR* hazard ratio, *PFS* progression-free survival, *OS* overall survival

At the 3-year follow-up, the median OS (95% CI) in the nivolumab group (5.26 [4.60–6.37] months) was longer than that in the placebo group (4.14 [3.42–4.86] months). The risk of death was significantly lower in the nivolumab group than in the placebo group (HR [95% CI], 0.62 [0.50–0.75], *P* < 0.0001; Fig. 1a). The OS rate was consistently higher in the nivolumab group than in the placebo group throughout the 3-year follow-up period. The 3-year OS rates were 5.6 and 1.9% in the nivolumab group and placebo group, respectively.

Median PFS (95% CI) was 1.61 (1.54–2.30) months in the nivolumab group and 1.45 (1.45–1.54) months in the placebo group at the 3-year follow-up (Fig. 1b). The risk of disease progression was lower in the nivolumab group than in the placebo group (HR [95% CI], 0.60 [0.49–0.75], *P* < 0.0001; Fig. 1b). PFS rates were consistently higher in the nivolumab group than in the placebo group after approximately 2 months from treatment initiation. The 3-year PFS rates were 2.4 and 0% in the nivolumab group and placebo group, respectively (Fig. 1b).

Among the 192 patients whose tumor tissues were available for biomarker analysis, we found no difference in the efficacy of nivolumab compared with that of placebo in the subgroup analysis of OS categorized by biomarkers such as PD-L1, TMB, and MSI status (Online Resource Table 1).

**Fig. 2:**
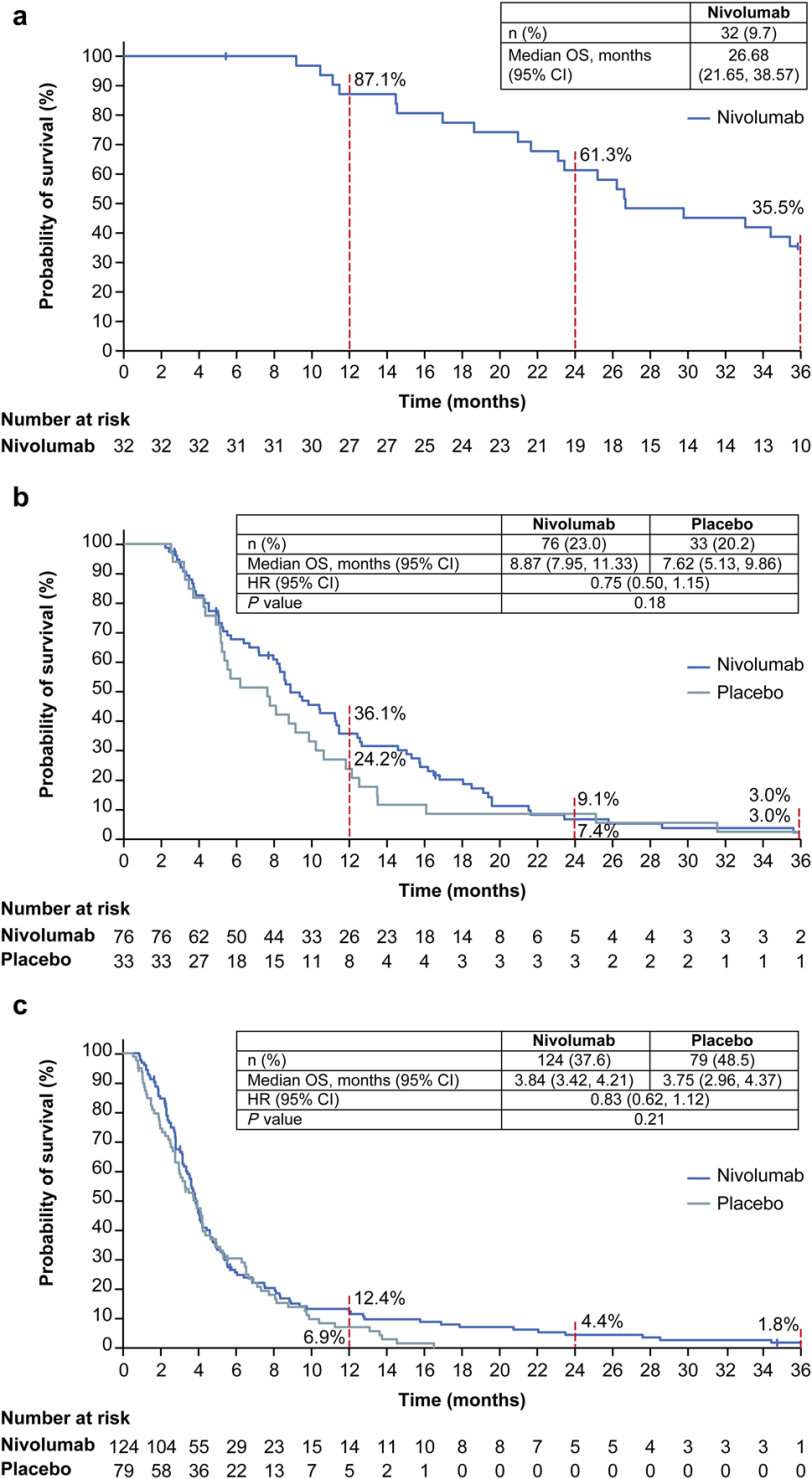
Subanalysis of OS by BOR among patients with CR + PR (**a**), SD (**b**), and PD (**c**). Vertical marks on the curve indicate patients who were censored. *BOR* best overall response, *CI* confidence interval, *CR* complete response, *HR* hazard ratio, *OS* overall survival, *PD* progressive disease, *PR* partial response, *SD* stable disease

The ORR and BOR were the same as reported at the 2-year follow-up [19]. No patient in the placebo group achieved CR or PR. Among 32 patients with CR or PR (responders) in the nivolumab group, the median DOR (95% CI) was 10.12 (8.31–16.72) months, and the median (95% CI) OS was 26.68 (21.65–38.57) months, with 1-year, 2-year, and 3-year OS rates of 87.1, 61.3, and 35.5%, respectively (Fig. 2a). All three patients with CR on nivolumab treatment survived longer than 3 years. The OS in patients with SD as their BOR was numerically longer in the nivolumab group vs the placebo group (median OS 8.87 vs 7.62 months; HR [95% CI], 0.75 [0.50–1.15], *P* = 0.18) (Fig. 2b). In patients with PD, the KM curves of the two treatment groups overlapped up to approximately 10 months before separating (median OS 3.84 vs 3.75 months; HR [95% CI], 0.83 [0.62–1.12], *P* = 0.21) (Fig. 2c).

The treatment duration of 3-year survivors receiving nivolumab and placebo is shown in Online Resource Fig. 1. In the nivolumab group, five of fifteen 3-year survivors received nivolumab for 3 years. In the placebo group, two of three 3-year survivors received nivolumab as subsequent therapy (data not shown).

### PPS in patients with or without TBP

**Fig. 3:**
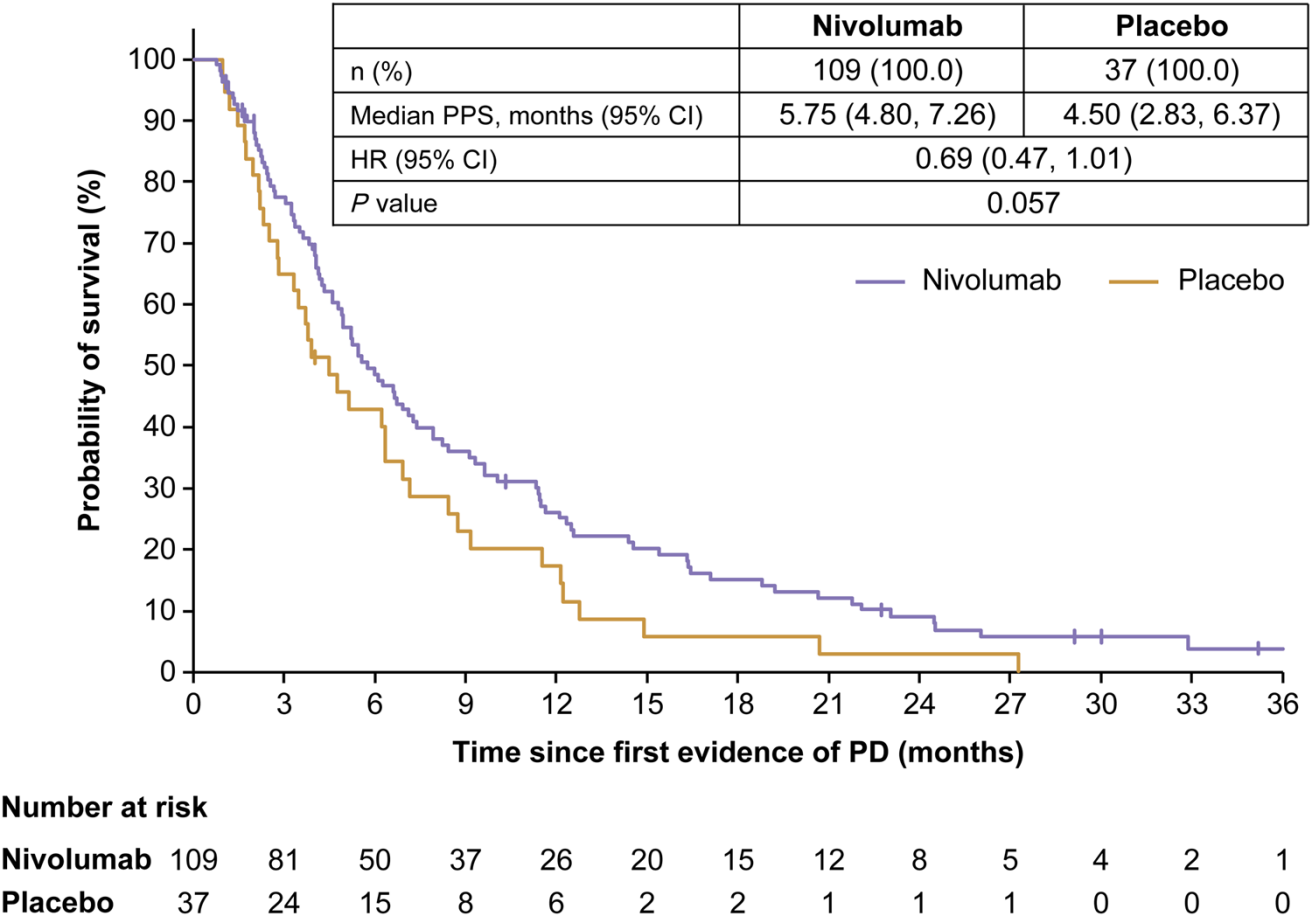
PPS in TBP patients after 3 years of follow-up. Vertical marks on the curve indicate patients who were censored. *CI* confidence interval, *HR* hazard ratio, *PD* progressive disease, *PPS* postprogression survival, *TBP* treatment beyond progression

Excluding the 38 and 29 patients who died before PD was determined, 20 and 17 patients in whom study drug administration was terminated due to apparent worsening of symptoms, 4 and 2 patients who could not be followed up after PD was determined, 3 and 2 patients in whom study drug administration was terminated due to adverse event(s), 4 and no patients in whom tumor shrinkage was persistent and continued at the 3-year follow-up, and 4 and no patients who were not determined to have PD due to other reasons from the nivolumab and placebo groups, respectively, the TBP cohort comprised 38.9% (109/280) and 28% (37/132) of patients with confirmation of the first event of PD in the nivolumab and placebo groups (Online Resource Fig. 2). Patient demographics and baseline characteristics were similar between the nivolumab and placebo groups both in the TBP and non-TBP patients (Table 1 and Online Resource Table 2). The median (range) duration of TBP was 1.12 (0–36.5) months and 1.08 (0–11.1) months in the nivolumab and placebo groups, respectively (Online Resource Table 3). The proportion of patients who received subsequent pharmacotherapy after nivolumab treatment among TBP patients (nivolumab group, 50.5% and placebo group, 54.1%; Table 1) was slightly higher than that among non-TBP patients (nivolumab group, 43.3% and placebo group, 34.7%; Online Resource Table 2). Among TBP patients, PPS tended to be longer in the nivolumab group (median PPS [95% CI], 5.75 [4.80–7.26] months) than in the placebo group (4.50 [2.83–6.37] months) with an HR (95% CI) of 0.69 (0.47–1.01) (*P* = 0.057) (Fig. 3). In contrast, among non-TBP patients, PPS was similar between the nivolumab and placebo groups (median PPS [95% CI], 2.27 [2.00–2.60] months vs 2.23 [1.58–2.69] months, respectively; HR [95% CI], 0.90 [0.70–1.16]; *P* = 0.42) (Online Resource Fig. 3).

**Table 1:** Patient demographics and baseline characteristics of patients receiving TBP

Parameter (unit)	Patients treated beyond progression		*P* value
	Nivolumab *n* (%)	Placebo *n* (%)	
*N*	109	37	
Sex			
Male	78 (71.6)	30 (81.1)	0.29
Female	31 (28.4)	7 (18.9)	
Age (years)			
< 65	58 (53.2)	20 (54.1)	1.00
≥ 65	51 (46.8)	17 (45.9)	
ECOG performance status score (eCRF source)			
0	38 (34.9)	15 (40.5)	0.56
1	71 (65.1)	22 (59.5)	
Recurrent			
No	58 (53.2)	23 (62.2)	0.44
Yes	51 (46.8)	14 (37.8)	
Histological type (Lauren classification)			
Intestinal type	43 (39.4)	18 (48.6)	0.25
Diffuse type	29 (26.6)	12 (32.4)	
Others	8 (7.3)	0	
Unknown	29 (26.6)	7 (18.9)	
Number of organs with metastases			
< 2	38 (34.9)	14 (37.8)	0.85
≥ 2	71 (65.1)	23 (62.2)	
Number of prior regimens			
2	15 (13.8)	8 (21.6)	0.38
3	48 (44.0)	12 (32.4)	
≥ 4	46 (42.2)	17 (45.9)	
PD-L1 expression			
≥ 1%	7 (6.4)	1 (2.7)	0.85
< 1%	38 (34.9)	13 (35.1)	
Missing	64 (58.7)	23 (62.2)	
Diameters of target lesions (mm)			
* n*	92	31	
Median	57.6	51.0	0.93
Time to first progression (months)			
Median	1.58	1.48	0.18
Mean	4.54	3.19	0.16
Poststudy treatment (pharmacotherapy)			
Yes	55 (50.5)	20 (54.1)	0.85
Fluoropyrimidine	21 (19.3)	10 (27.0)	
Taxane	15 (13.8)	7 (18.9)	
Platinum	13 (11.9)	7 (18.9)	
Irinotecan	9 (8.3)	3 (8.1)	
Ramucirumab	26 (23.9)	7 (18.9)	
Immunotherapy	3 (2.8)	1 (2.7)	
Other targeted therapies	3 (2.8)	0	
BOR			
CR	0	0	0.038
PR	14 (12.8)	0	
SD	27 (24.8)	7 (18.9)	
PD	48 (44.0)	24 (64.9)	
NE	20 (18.3)	6 (16.2)	

*BOR* best overall response, *CR* complete response, *ECOG* Eastern Cooperative Oncology Group, *eCRF* electronic case report form, *NE* not evaluated, *PD* progressive disease, *PD-L1* programmed death-ligand 1, *PR* partial response, *SD* stable disease, *TBP* treatment beyond progression

### Subanalysis of PPS by BOR in TBP patients

**Fig. 4:**
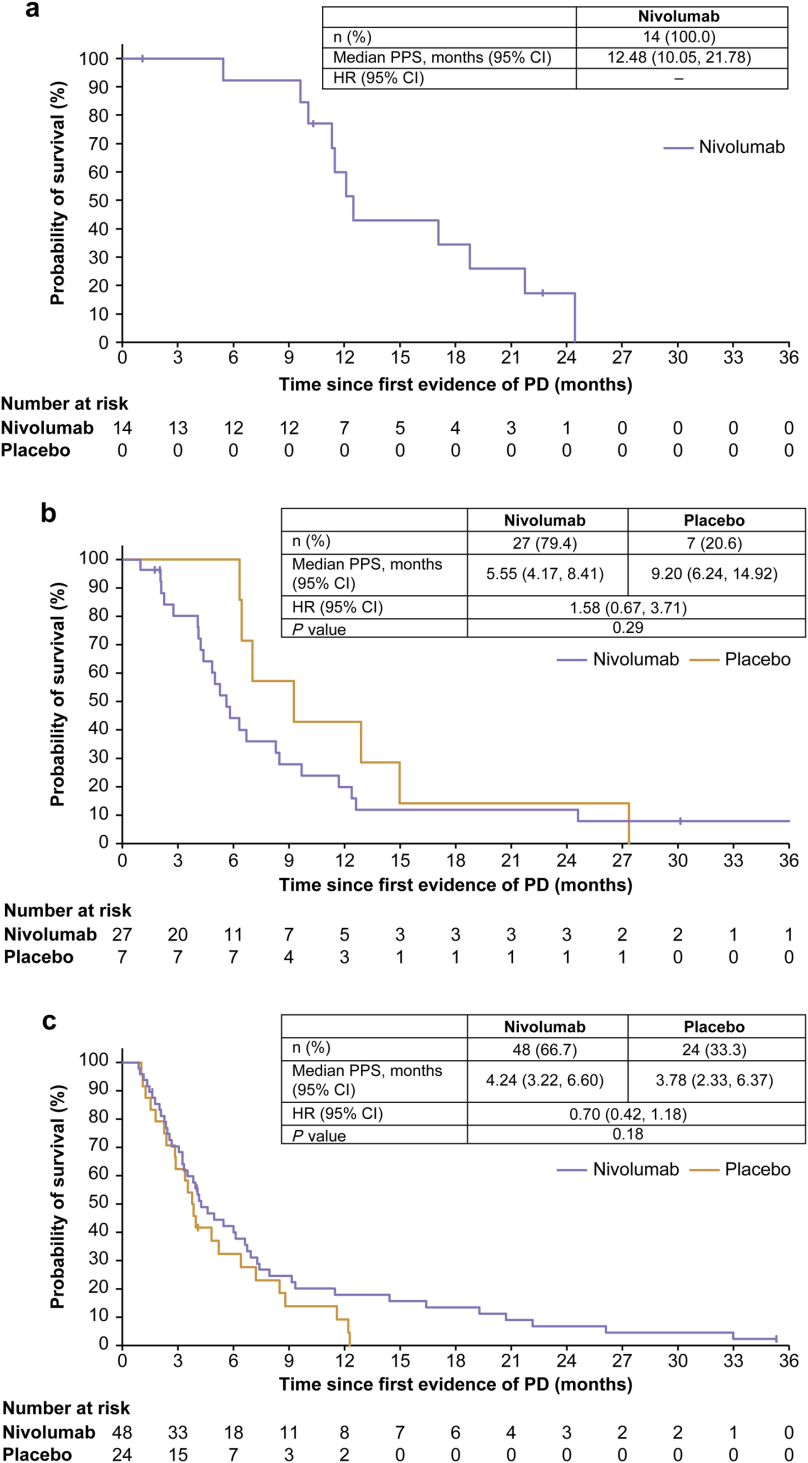
Subanalysis of PPS by BOR among TBP patients with CR + PR (**a**), SD (**b**), and PD (**c**). Vertical marks on the curve indicate patients who were censored. *BOR* best overall response, *CI* confidence interval, *CR* complete response, *HR* hazard ratio, *PD* progressive disease, *PPS* postprogression survival, *PR* partial response, *SD* stable disease, *TBP* treatment beyond progression

Among TBP patients, the median (95% CI) PPS was 12.48 (10.05–21.78) months in patients achieving CR or PR in the nivolumab group (Fig. 4a) with a median (range) TBP duration of 4.75 (0.2–10.9) months (Online Resource Table 3). In patients with the BOR of SD in the nivolumab and placebo groups, the median (range) TBP duration was 1.12 (0–36.5) and 1.45 (0–11.1) months and the median (95% CI) PPS was 5.55 (4.17–8.41) months and 9.20 (6.24–14.92) months, respectively (HR [95% CI], 1.58 [0.67–3.71]) (Fig. 4b). In patients with the BOR of PD, the median TBP duration was 1.05 (0–11.0) and 1.08 (0–2.6) months, and PPS was similar until the median (95% CI) between the nivolumab group (4.24 [3.22–6.60] months) and the placebo group (3.78 [2.33–6.37] months); thereafter, the nivolumab group showed favorable long-term survival compared with the placebo group (HR [95% CI], 0.70 [0.42–1.18]) (Fig. 4c).

### Change in tumor size in TBP patients

Among 109 patients receiving TBP with nivolumab, 89 patients had measurable lesions and imaging data for evaluation of the first event of PD. After excluding 17 patients who did not have imaging data and/or meet the criteria of PD defined in RECIST (increase in tumor size by ≥ 20% or appearance of new lesions) from these 89 patients, 72 patients had measurable lesions and confirmation of the first event of PD by imaging. Their PD patterns were an increase in the tumor size of existing target lesions by ≥ 20% without new lesions in 24 patients, increase in tumor size by < 20% with new lesions in 31 patients, and increase in tumor size by ≥ 20% with new lesions in 17 patients. According to the BOR of these 72 patients, PD pattern due to appearance of new lesion with increase in tumor size by < 20% was observed in 8 (80%) of 10 patients with BOR of CR/PR, 9 (50%) of 18 patients with SD, and 14 (32%) of 44 with PD (Online Resource Fig. 2).

**Fig. 5:**
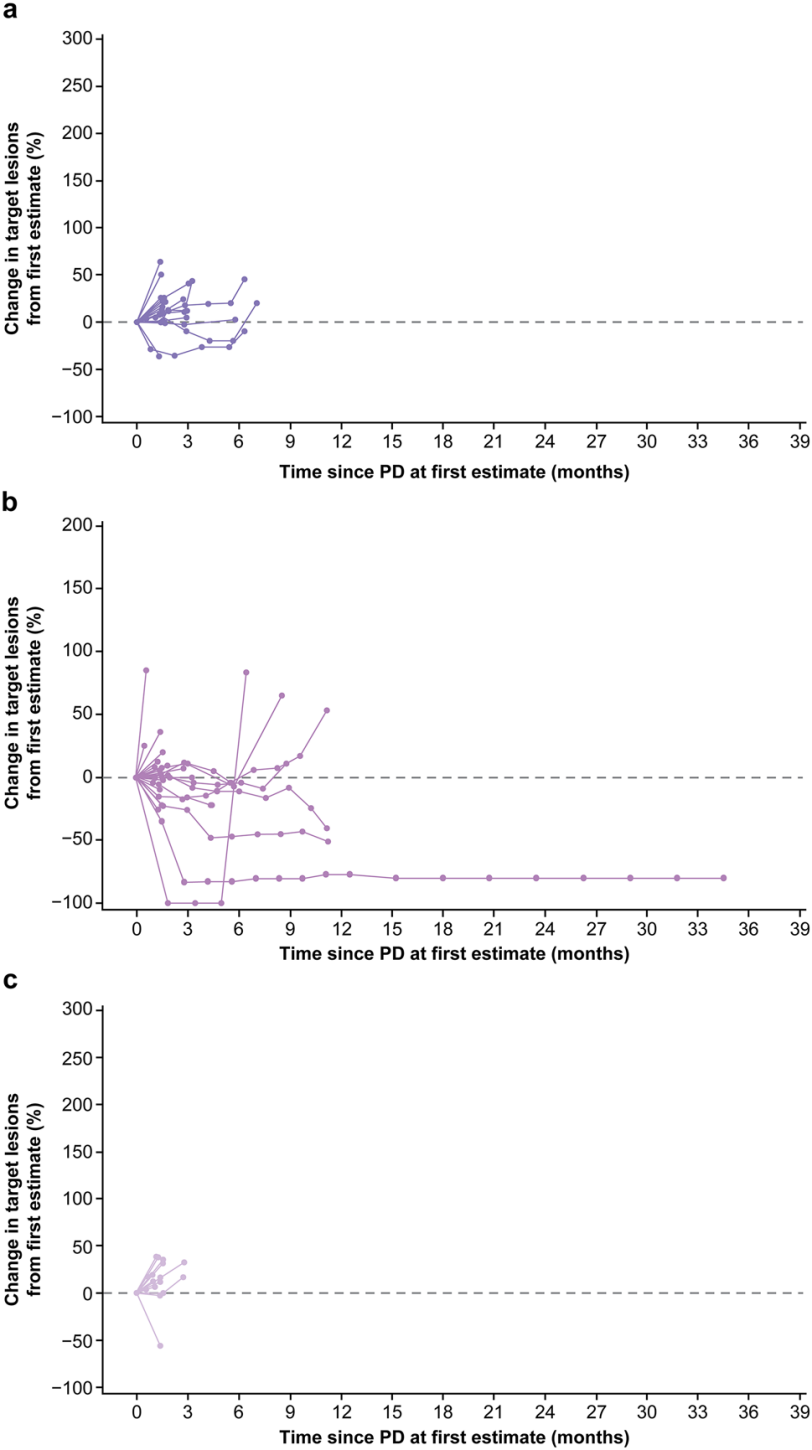
Spider plot during TBP of patients who were evaluated for BOR in the nivolumab group, categorized by PD patterns, in patients with target lesion. Spider plot showing PD categorized by PD patterns in TBP patients with target lesion progression of ≥ 20% without new lesions (*n* = 24) (**a**), target lesion progression of < 20% with appearance of new lesions (*n* = 31) (**b**), and target lesion progression of ≥ 20% and appearance of new lesions (*n* = 17) (**c**). *BOR* best overall response, *PD* progressive disease, *TBP* treatment beyond progression

Figure 5 shows a spider plot during TBP with nivolumab in these 72 patients categorized by PD patterns—target lesion progression of ≥ 20% without a new lesion (*n* = 24, Fig. 5a), target lesion progression of < 20% but appearance of a new lesion (*n* = 31, Fig. 5b), and both a target lesion progression of ≥ 20% and a new lesion (*n* = 17, Fig. 5c). During TBP, some tumor shrinkage compared with the tumor size at the first event of PD was observed only in 7 of 41 (17%) patients with progression of target lesion by ≥ 20% regardless of appearance of a new lesion. On the contrary, 15 of 31 (48%) patients with lesion progression of < 20% in previously existing tumor lesions and new lesion emergence had some tumor shrinkage during TBP.

In patients with the BOR of CR/PR (*n* = 8), SD (*n* = 9), and PD (*n* = 14) who experienced PD due to the appearance of a new lesion without progression of target lesion by ≥ 20%, 6 (75%), 2 (22%), and 7 (50%) patients, respectively, had some tumor shrinkage during TBP (Online Resource Figs. 4, 5, and 6).

### Safety

No new TRAEs developed after the previously reported 2-year follow-up [19]. In the nivolumab group, TRAEs of special interest were interstitial lung disease (*n* = 6 [1.8%]), maculopapular rash (*n* = 5 [1.5%]), colitis (*n* = 2 [0.6%]), hyperthyroidism (*n* = 2 [0.6%]), pneumonitis (*n* = 2 [0.6%]), acute hepatitis (*n* = 1 [0.3%]), autoimmune thyroiditis (*n* = 1 [0.3%]), hypopituitarism (*n* = 1 [0.3%]), and thyroid disorder (*n* = 1 [0.3%]) at the 3-year follow-up. Maculopapular rash was observed in one patient of the placebo group (Online Resource Table 4).

## Discussion

The 3-year follow-up of the ATTRACTION-2 study confirmed that nivolumab consistently prolonged the OS compared with placebo and was associated with numerically higher 3-year OS and PFS rates in patients with unresectable advanced or recurrent G/GEJ cancer after failure of ≥ 2 prior chemotherapy regimens. A total of 15 patients in the nivolumab group and three patients in the placebo group survived longer than 3 years (two of the three 3-year survivors in the placebo group received nivolumab as subsequent therapy). Noticeably, responders with PR or CR in the nivolumab group had a favorable 3-year survival rate, as high as 35.5%. Moreover, among the patients with SD and PD as their BOR, treatment with nivolumab resulted in relatively longer survival compared with placebo. Although the overall long-term survival rate was not satisfactory compared with that in melanoma and lung cancer, it is considered that nivolumab can contribute to prolongation of survival in some patients with advanced or recurrent G/GEJ regardless of the BOR [8, 15–18].

In renal cell carcinoma (CheckMate 025) [27] and head and neck cancer (CheckMate 141) [31], TBP with nivolumab had a survival benefit. However, as a limitation of these analyses, patients who received TBP might be in a better condition compared with those who did not. Medical condition at initiation of pharmacotherapy influences its efficacy. Indeed, the demographics and background characteristics of patients in this study showed that the proportions of patients with poor prognostic factors, such as ECOG PS 1, number of metastatic organs ≥ 2, and large diameter of target lesions, were relatively lower in the TBP cohort compared with the non-TBP cohort. However, there were no remarkable differences in patient background at enrollment between the nivolumab and placebo groups in the TBP cohort (Table 1), while there were some differences in the BOR before starting TBP. Among the TBP patients, PPS tended to be longer in the nivolumab group than in the placebo group (median 5.75 months vs 4.50 months; HR 0.69), while PPS was similar between them among the non-TBP patients (median 2.27 months vs 2.23 months, respectively; HR 0.90). This subanalysis suggests the efficacy of TBP with nivolumab over placebo for unresectable advanced or recurrent G/GEJ cancer. However, the median duration of TBP with nivolumab was as short as 1.12 months, which means that TBP was discontinued due to a second PD at the first evaluation after initiating TBP in more than half of the patients. Thus, it is not clear which could provide better clinical outcomes, TBP with nivolumab or a switch to other pharmacotherapies. The clinical decision for selecting optimal treatments at the first event of PD with nivolumab is important for patients with advanced unresectable or recurrent G/GEJ cancer who have small chances for further treatment. Thus, it is necessary to establish biomarkers for identifying patients suitable for TBP with nivolumab.

Patients who received TBP after the first event of PD included patients with various BORs such as CR, PR, SD, and PD. The median (95% CI) PPS of responders in the nivolumab group was as long as 12.48 (10.05–21.78) months (Fig. 4a) with a relatively long median TBP duration of 4.75 months. This median TBP duration appeared to be longer than the median PFS of irinotecan [32] and trifluridine–tipiracil [33]. However, the extent of contribution of nivolumab during TBP toward a favorable PPS in these responders is not clear since the patients’ condition could be improved by response to nivolumab before starting TBP. Furthermore, the immunological status modified by nivolumab might have a good influence on the efficacy of subsequent chemotherapy. Although it is still unclear which is beneficial, i.e., TBP with nivolumab or switching to subsequent chemotherapy after the first event of PD even for responders, responders might be candidates for TBP with nivolumab.

Unexpectedly, among TBP patients with the BOR of SD, the placebo group tended to have a longer PPS compared with the nivolumab group. This observation could be attributed to the small sample size of TBP patients with the BOR of SD, particularly in the placebo group (only 7 patients). Furthermore, it is speculated that patients with the BOR of SD in the placebo group had naturally indolent tumors, with a range of TBP duration with placebo from 0 to 11.1 months, whereas some patients with SD in the nivolumab group might have had an originally aggressive tumor whose progression was suppressed by nivolumab. The criteria for SD range from shrinkage by < 30% to growth by < 20%, and some tumor shrinkage by < 30% within SD represents the efficacy of nivolumab. In fact, in our previous report, patients achieving tumor shrinkage by 5–30% showed longer survival and a better HR than those with tumor growth by 5–20% [19]. Thus, among patients with the BOR of SD who received TBP, there might be some differences in tumor biology between the nivolumab and placebo groups, and there was a substantial variation in the response to nivolumab. These factors should be taken into consideration when deciding TBP with nivolumab for patients with the BOR of SD.

Among TBP patients with the BOR of PD, the nivolumab group showed a relatively longer PPS compared with the placebo group (4.24 months vs 3.78 months; HR 0.70). In the nivolumab group, 10 of 14 1-year survivors with the BOR of PD received TBP (Fig. 2c). In contrast, among patients with the BOR of PD who did not receive TBP, there was no difference in PPS between the nivolumab group and the placebo group (Online Resource Fig. 7). These results suggest that TBP with nivolumab might have a survival benefit compared with placebo even for patients with the BOR of PD if the patient’s condition is preserved. However, this subgroup did not include patients experiencing rapid progression for which TBP was judged to be inappropriate by physicians. It should be noted that TBP with nivolumab should not be performed after rapid progression (not allowed in the protocol of the ATTRACTION-2 study).

In some cases, conventional guidelines for response assessment do not work well for the evaluation of the tumor response to immunotherapy. Recently, a new guideline, iRECIST, has been proposed specifically for immunotherapy [34]. In iRECIST, the appearance of a new lesion is not classified as PD but is recognized as a new evaluable or non-evaluable lesion that is added to pre-existing lesions for response assessment [34]. In this study, TBP was evaluated according to the PD pattern per RECIST (tumor progression of previously existing lesions and/or new lesion emergence). Among 41 patients with progression of target lesion by ≥ 20% with or without new lesions, 7 (17%) patients had some tumor shrinkage during TBP. Although a common consensus on the definition of pseudoprogression is still lacking [25, 35], the incidence of pseudoprogression is considered to be rare in solid tumors [36]. In contrast, many of the responders (8/10, 80%) experienced PD due to the appearance of a new lesion without progression of existing target lesions by ≥ 20% and showed favorable clinical outcome in TBP with nivolumab. Among all patients (*n* = 72) evaluated with PD pattern regardless of BOR, 31 (44%) experienced PD due to the appearance of a new lesion without progression of target lesion by ≥ 20%. In these 31 patients, 15 (48%) had some tumor shrinkage associated with long duration of TBP. Thus, regardless of BOR, patients who experienced PD due to the appearance of a new lesion without progression of ≥ 20% in previously existing target lesions had a trend to have longer PPS with nivolumab than those who had other PD patterns. These results suggest that the immunological environment differs depending on the disease sites (pattern of PD) and that TBP with nivolumab might be effective in patients with lesion progression < 20% in previously existing target lesions and new lesion emergence. It is expected that future biomarker research based on these results will lead to finding biomarkers not only for identifying patients suitable for TBP with nivolumab but also for new combination therapy with other immunological drugs and/or molecular targeted agents.

This study has some limitations. This was a post hoc analysis of the TBP subsets with a small sample size. Information on patient background characteristics at the start of TBP was not collected. Translational research for biomarkers predicting the efficacy of nivolumab in TBP patients will be required to confirm these observations.

## Conclusions

The long-term efficacy of nivolumab was confirmed throughout the 3-year follow-up period in the ATTRACTION-2 study, with no new safety signals. TBP with nivolumab showed a tendency to prolong PPS compared with placebo, and a survival benefit of TBP with nivolumab was suggested. The clinical significance of TBP with nivolumab and biomarkers for selecting patients suitable for TBP should be verified in the near future.

## Supplementary information

Below is the link to the electronic supplementary material.

Supplementary file1 (DOC 1294 KB)

## References

[1] Sanghera C, Sanghera R (2019). Immunotherapy—strategies for expanding its role in the treatment of all major tumor sites. Cureus.

[2] Hodi FS, O’Day SJ, McDermott DF, Weber RW, Sosman JA, Haanen JB (2010). Improved survival with ipilimumab in patients with metastatic melanoma. N Engl J Med.

[3] Robert C, Long GV, Brady B, Dutriaux C, Maio M, Mortier L (2015). Nivolumab in previously untreated melanoma without BRAF mutation. N Engl J Med.

[4] Ferris RL, Blumenschein G, Fayette J, Guigay J, Colevas AD, Licitra L (2016). Nivolumab for recurrent squamous-cell carcinoma of the head and neck. N Engl J Med.

[5] Kiyota N, Hasegawa Y, Takahashi S, Yokota T, Yen CJ, Iwae S (2017). A randomized, open-label, Phase III clinical trial of nivolumab vs. therapy of investigator’s choice in recurrent squamous cell carcinoma of the head and neck: a subanalysis of Asian patients versus the global population in Checkmate 141. Oral Oncol.

[6] Tomita Y, Fukasawa S, Shinohara N, Kitamura H, Oya M, Eto M (2019). Nivolumab versus everolimus in advanced renal cell carcinoma: Japanese subgroup 3-year follow-up analysis from the Phase III CheckMate 025 study. Jpn J Clin Oncol.

[7] Brahmer J, Reckamp KL, Baas P, Crinò L, Eberhardt WE, Poddubskaya E (2015). Nivolumab versus docetaxel in advanced squamous-cell non-small-cell lung cancer. N Engl J Med.

[8] Chen R, Tao Y, Xu X, Shan L, Jiang H, Yin Q (2018). The efficacy and safety of nivolumab, pembrolizumab, and atezolizumab in treatment of advanced non-small cell lung cancer. Discov Med.

[9] Kang YK, Boku N, Satoh T, Ryu MH, Chao Y, Kato K (2017). Nivolumab in patients with advanced gastric or gastro-oesophageal junction cancer refractory to, or intolerant of, at least two previous chemotherapy regimens (ONO-4538-12, ATTRACTION-2): a randomised, double-blind, placebo-controlled, phase 3 trial. Lancet.

[10] Bang YJ, Ruiz EY, Van Cutsem E, Lee KW, Wyrwicz L, Schenker M (2018). Phase III, randomised trial of avelumab versus physician’s choice of chemotherapy as third-line treatment of patients with advanced gastric or gastro-oesophageal junction cancer: primary analysis of JAVELIN Gastric 300. Ann Oncol.

[11] Shitara K, Özgüroğlu M, Bang YJ, Di Bartolomeo M, Mandalà M, Ryu MH (2018). Pembrolizumab versus paclitaxel for previously treated, advanced gastric or gastro-oesophageal junction cancer (KEYNOTE-061): a randomised, open-label, controlled, phase 3 trial. Lancet.

[12] Shitara K, Van Cutsem E, Bang YJ, Fuchs C, Wyrwicz L, Lee KW (2020). Efficacy and safety of pembrolizumab or pembrolizumab plus chemotherapy vs chemotherapy alone for patients with first-line, advanced gastric cancer: the KEYNOTE-062 phase 3 randomized clinical trial. JAMA Oncol..

[13] Boku N, Ryu MH, Oh D-Y, Chung HC, Lee KW, Omori T, *et al*. Nivolumab plus chemotherapy versus chemotherapy alone in patients with previously untreated advanced or recurrent gastric/gastroesophageal junction (G/GEJ) cancer: ATTRACTION-4 (ONO-4538-37) study. In: Oral presentation at European Society for Medical Oncology virtual congress. 2020, Abstr LBA7.

[14] Moehler M, Shitara K, Garrido M, Salman P, Shen L, Wyrwicz L, *et al*. Nivolumab (NIVO) plus chemotherapy (chemo) versus chemo as first-line (1L) treatment for advanced gastric cancer/gastroesophageal junction cancer (GC/GEJC)/esophageal adenocarcinoma (EAC): first results of the CheckMate 649 study. In: Oral presentation at European Society for Medical Oncology virtual congress. 2020, Abstr LBA6.

[15] Yamazaki N, Kiyohara Y, Uhara H, Uehara J, Fujisawa Y, Takenouchi T (2019). Long-term follow up of nivolumab in previously untreated Japanese patients with advanced or recurrent malignant melanoma. Cancer Sci.

[16] Ascierto PA, Long GV, Robert C, Brady B, Dutriaux C, Di Giacomo AM (2019). Survival outcomes in patients with previously untreated BRAF wild-type advanced melanoma treated with nivolumab therapy: three-year follow-up of a randomized phase 3 trial. JAMA Oncol.

[17] Rogiers A, Boekhout A, Schwarze JK, Awada G, Blank CU, Neyns B (2019). Long-term survival, quality of life, and psychosocial outcomes in advanced melanoma patients treated with immune checkpoint inhibitors. J Oncol.

[18] Gettinger S, Horn L, Jackman D, Spigel D, Antonia S, Hellmann M (2018). Five-year follow-up of nivolumab in previously treated advanced non-small-cell lung cancer: results from the CA209-003 study. J Clin Oncol.

[19] Chen LT, Satoh T, Ryu MH, Chao Y, Kato K, Chung HC (2020). A phase 3 study of nivolumab in previously treated advanced gastric or gastroesophageal junction cancer (ATTRACTION-2): 2-year update data. Gastric Cancer.

[20] Wainberg ZA, Yoon HH, Catenacci DVT, Jalal SI, Muro K, Garrido M (2019). Efficacy and safety of pembrolizumab (pembro) alone or in combination with chemotherapy (chemo) in patients (pts) with advanced gastric or gastroesophageal (G/GEJ) cancer: long-term follow up from KEYNOTE-059. J Clin Oncol..

[21] Onesti CE, Frères P, Jerusalem G (2019). Atypical patterns of response to immune checkpoint inhibitors: interpreting pseudoprogression and hyperprogression in decision making for patients’ treatment. J Thorac Dis.

[22] Soria F, Beleni AI, D'Andrea D, Resch I, Gust KM, Gontero P (2018). Pseudoprogression and hyperprogression during immune checkpoint inhibitor therapy for urothelial and kidney cancer. World J Urol.

[23] Raimondi A, Randon G, Sepe P, Claps M, Verzoni E, de Braud F (2019). The evaluation of response to immunotherapy in metastatic renal cell carcinoma: open challenges in the clinical practice. Int J Mol Sci.

[24] Rini BI, Battle D, Figlin RA, George DJ, Hammers H, Hutson T (2019). The society for immunotherapy of cancer consensus statement on immunotherapy for the treatment of advanced renal cell carcinoma (RCC). J Immunother Cancer.

[25] Borcoman E, Nandikolla A, Long G, Goel S, Le Tourneau C (2018). Patterns of response and progression to immunotherapy. Am Soc Clin Oncol Educ Book.

[26] Wang Q, Gao J, Wu X (2018). Pseudoprogression and hyperprogression after checkpoint blockade. Int Immunopharmacol.

[27] Escudier B, Motzer RJ, Sharma P, Wagstaff J, Plimack ER, Hammers HJ (2017). Treatment beyond progression in patients with advanced renal cell carcinoma treated with nivolumab in CheckMate 025. Eur Urol.

[28] Long GV, Weber JS, Larkin J, Atkinson V, Grob JJ, Schadendorf D (2017). Nivolumab for patients with advanced melanoma treated beyond progression: analysis of 2 Phase 3 clinical trials. JAMA Oncol.

[29] Ricciuti B, Genova C, Bassanelli M, De Giglio A, Brambilla M, Metro G (2019). Safety and efficacy of nivolumab in patients with advanced non-small-cell lung cancer treated beyond progression. Clin Lung Cancer.

[30] Eisenhauer EA, Therasse P, Bogaerts J, Schwartz LH, Sargent D, Ford R (2009). New response evaluation criteria in solid tumours: revised RECIST guideline (version 1.1). Eur J Cancer.

[31] Haddad R, Concha-Benavente F, Blumenschein G, Fayette J, Guigay J, Colevas AD (2019). Nivolumab treatment beyond RECIST-defined progression in recurrent or metastatic squamous cell carcinoma of the head and neck in CheckMate 141: a subgroup analysis of a randomized phase 3 clinical trial. Cancer.

[32] Ishii T, Kawazoe A, Sasaki A, Mishima S, Kentaro S, Nakamura Y, *et al*. Clinical and molecular factors for selection of nivolumab or irinotecan as third-line treatment for advanced gastric cancer. Ther Adv Med Oncol. 2020;12. 10.1177/1758835920942377 PMC7370559 32733607

[33] Shitara K, Doi T, Dvorkin M, Mansoor W, Arkenau HT, Prokharau A (2018). Trifluridine/tipiracil versus placebo in patients with heavily pretreated metastatic gastric cancer (TAGS): a randomised, double-blind, placebo-controlled, phase 3 trial. Lancet Oncol.

[34] Persigehl T, Lennartz S, Schwartz LH (2020). iRECIST: how to do it. Cancer Imaging.

[35] Wolchok JD, Hoos A, O’Day S, Weber JS, Hamid O, Lebbé C (2009). Guidelines for the evaluation of immune therapy activity in solid tumors: immune-related response criteria. Clin Cancer Res.

[36] Chiou VL, Burotto M (2015). Pseudoprogression and immune-related response in solid tumors. J Clin Oncol.

