# Supplementary material for: Nivolumab in previously treated advanced gastric cancer (ATTRACTION-2): 3-year update and outcome of treatment beyond progression with nivolumab
Source: Gastric Cancer. 2021 Mar 20;24(4):946–58. doi: 10.1007/s10120-021-01173-w (PMC8205916; doi:10.1007/s10120-021-01173-w)
Supplement: Supplementary file 1 — Supplementary file1 (DOC 1294 KB) [file 10120_2021_1173_MOESM1_ESM.doc]

**Supplementary section**

**Online Resource Fig. 1** Overall duration of nivolumab and placebo treatment among patients who survived for more than 3 years in ATTRACTION-2

*CR* complete response, *NE* not evaluated, *NIV* nivolumab, *PD* progressive disease, *PLA* placebo, *PR* partial response, *SD* stable disease

**Online Resource Fig. 2** Disposition of patients who received TBP


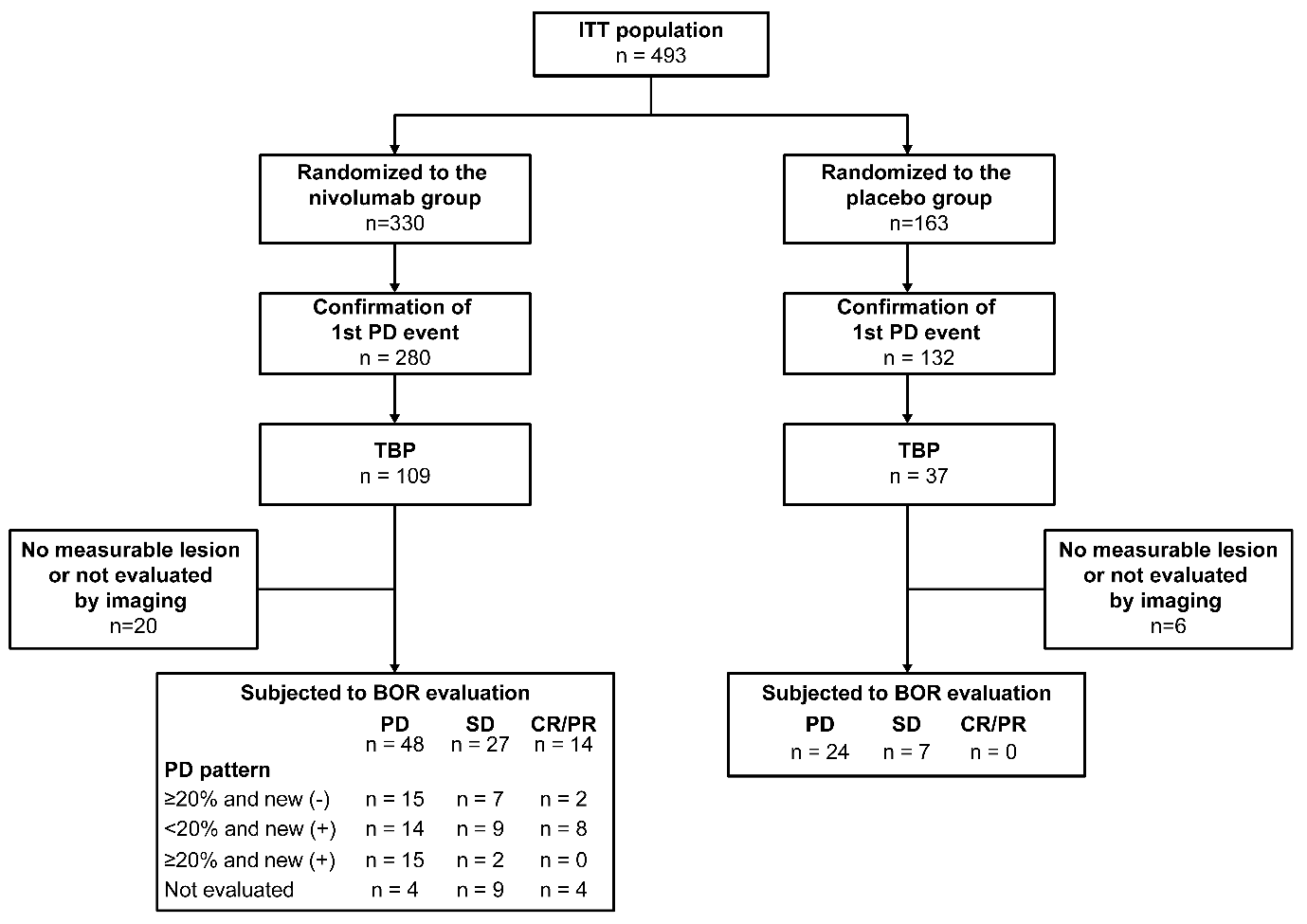


*BOR* best overall response, *CR* complete response, *ITT* intent to treat, *PD* progressive disease, *PR* partial response, *SD* stable disease, *TBP* treatment beyond progression

**Online Resource Fig. 3** Kaplan-Meier plot of PPS in patients who did not receive TBP during the 3 years of follow-up

Vertical marks on the curve indicate patients who were censored

*CI* confidence interval, *HR* hazard ratio, *PD* progressive disease, *PPS* postprogression survival, *TBP* treatment beyond progression

**Online Resource Fig. 4** Spider plot during TBP of patients showing BOR of CR or PR in the nivolumab group, categorized by PD patterns, in patients with target lesion progression of  ≥ 20% without new lesions (*n* = 2) (**a**) and in those with target lesion progression of < 20% with appearance of new lesions (*n* = 8) (**b**)


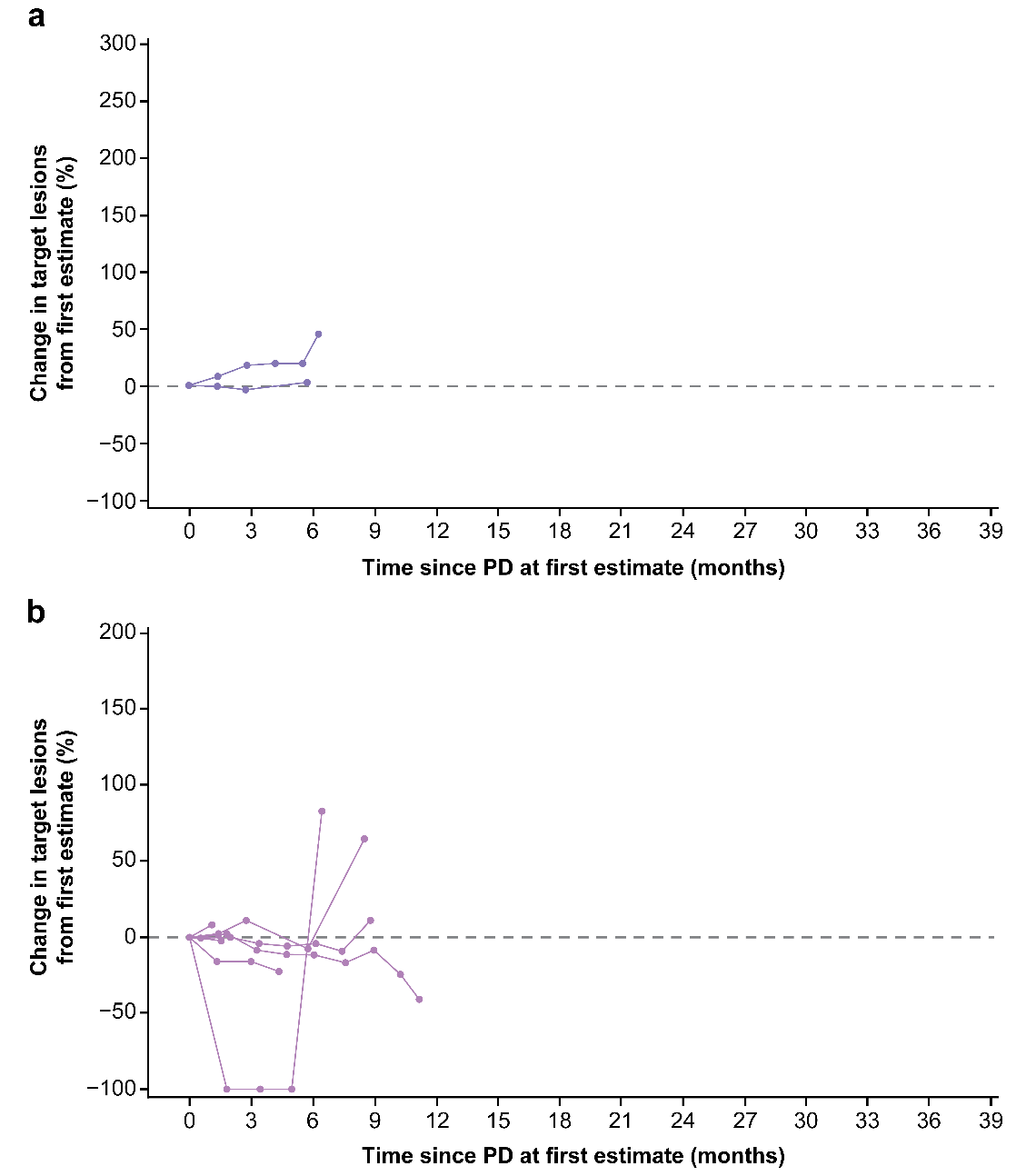


There was no patient with target lesion progression of ≥ 20% and none with appearance of new lesions among CR or PR patients who received TBP with nivolumab

One patient who received nivolumab two or more times after PD evaluation but did not have imaging data was included in (**b**)

*BOR* best overall response, *CR* complete response, *PD* progressive disease, *PR* partial response, *TBP* treatment beyond progression

**Online Resource Fig. 5** Spider plot during TBP of patients showing BOR of SD categorized by PD patterns of TBP patients in the nivolumab group with target lesion progression of ≥ 20% without new lesions (*n* = 7) (**a**), target lesion progression of < 20% with appearance of new lesions (*n* = 9) (**b**), and target lesion progression of ≥ 20% and appearance of new lesions (*n* = 2) (**c**)


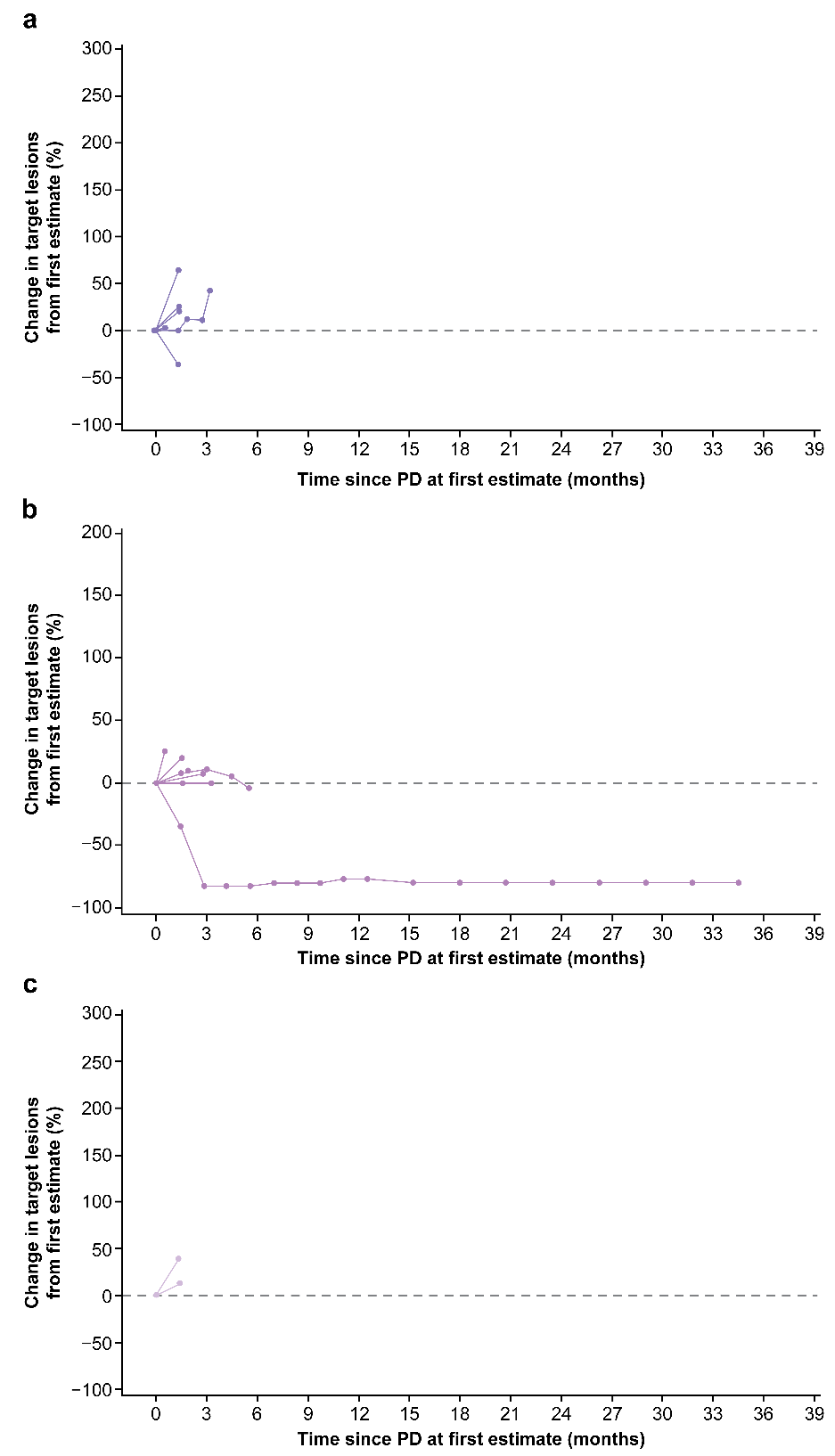


One patient who received nivolumab once on the same day of PD evaluation was included in (**a**)

One patient who received nivolumab once immediately after the day of PD evaluation was included in (**b**)

*BOR* best overall response, *PD* progressive disease, *SD* stable disease, *TBP* treatment beyond progression

**Online Resource Fig. 6** Spider plot during TBP of patients showing BOR of PD categorized by PD patterns of TBP patients in the nivolumab group with target lesion progression of ≥ 20% without new lesions (*n* = 15) (**a**), target lesion progression of < 20% with appearance of new lesions (*n* = 14) (**b**), and target lesion progression of ≥ 20% and appearance of new lesions (*n* = 15) (**c**)


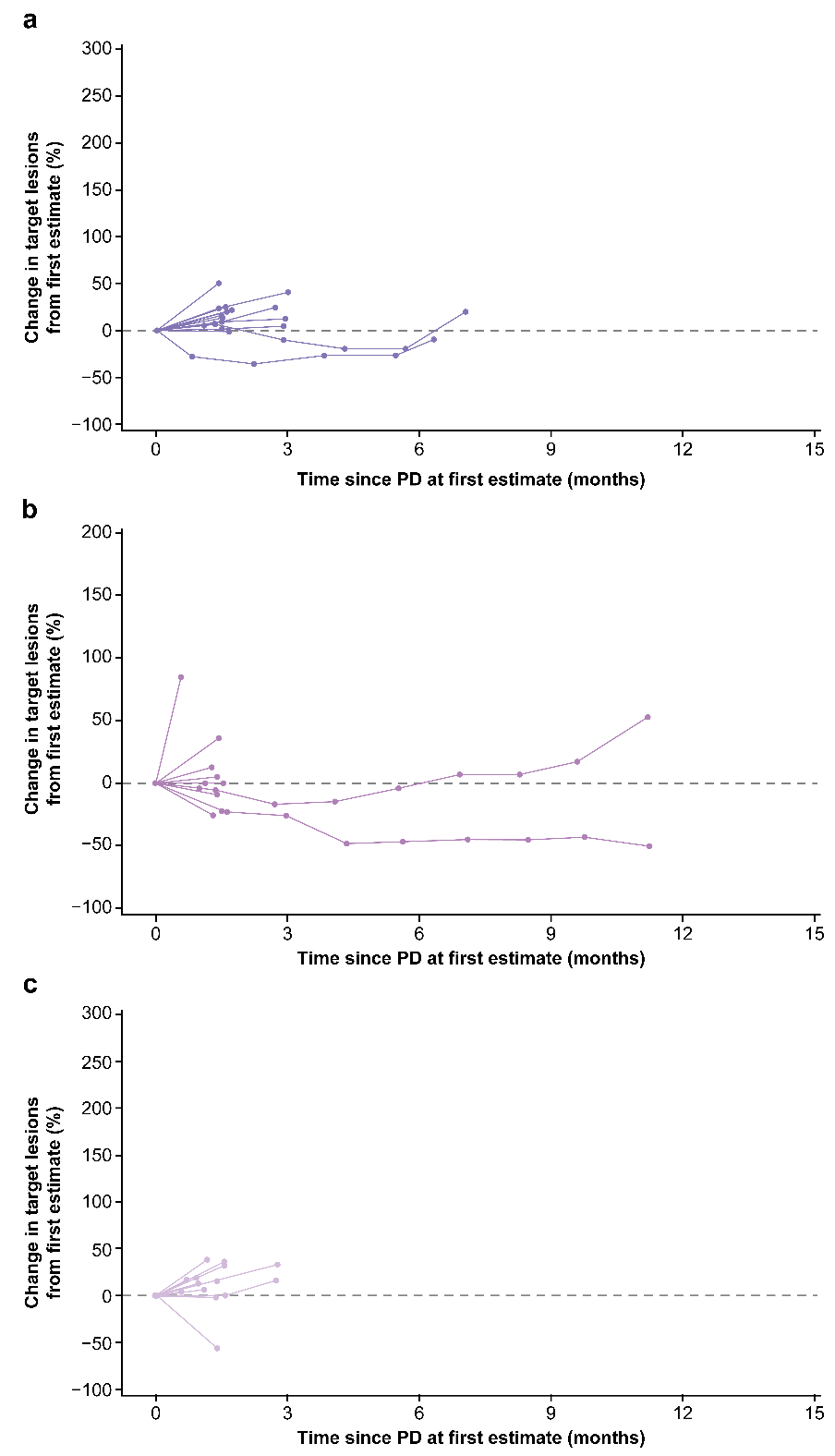


One patient each who received nivolumab two or more times after PD evaluation but did not have imaging data were included in (**a**) and (**c**), respectively

Two and two patients who received nivolumab once immediately after the day of PD evaluation were included in (**b**) and (**c**), respectively.

*BOR* best overall response, *PD* progressive disease, *TBP* treatment beyond progression

**Online Resource Fig. 7** Kaplan-Meier plot of PPS in patients with the BOR of PD who did not receive TBP during the 3 years of follow-up

 Vertical marks on the curve indicate patients who were censored

*BOR* best overall response, *CI* confidence interval, *HR* hazard ratio, *PD* progressive disease, *PPS* postprogression survival, *TBP* treatment beyond progression

**Online Resource Table 1** Tissue biomarker analysis

| **PD-L1 expression in tumor cell, *n* (%)** | ***n* = 130** | ***n* = 62** | HR |
| --- | --- | --- | --- |
|  |  |  | [95% CI] |
| < 1% | 114 (87.7) | 52 (83.9) | 0.66 |
|  |  |  | [0.47, 0.92] |
| ≥ 1% | 16 (12.3) | 10 (16.1) | 0.75 |
|  |  |  | [0.32, 1.72] |
| **TMB analysis, *n* (%)** | ***n* = 91** | ***n* = 45** |  |
| ≥ 0 and < 5 | 48 (52.7) | 23 (51.1) | 0.73 |
|  |  |  | [0.44, 1.23] |
| ≥ 5 and < 10 | 33 (36.3) | 14 (31.1) | 0.47 |
|  |  |  | [0.24, 0.94] |
| ≥ 10 | 9 (9.9) | 6 (13.3) | 0.52 |
|  |  |  | [0.16, 1.62] |
| Not detected | 1 (1.1) | 2 (4.4) | - |
| **MSI status, *n* (%)** | ***n* = 91** | ***n* = 45** | - |
| MSI-H | 1 (1.1) | 3 (6.7) | - |
| MSS | 82 (90.1) | 33 (73.3) | 0.61 |
|  |  |  | [0.40, 0.93] |
| Unknown | 8 (8.8) | 9 (20.0) | - |

*CI* confidence interval, *HR* hazard ratio, *MSI-H* microsatellite instability-high, *MSS* microsatellite stable, *PD-L1* programmed death-ligand 1, *TMB* tumor mutation burden

**Online Resource Table 2** Patient demographics and baseline characteristics of non-TBP patients

|  | **Patients not treated beyond progression** | |
| --- | --- | --- |
| **Parameter (unit)** | **Nivolumab**  ***n* (%)** | **Placebo**  ***n* (%)** |
| *N* | 171 | 95 |
| Sex |  |  |
| Male | 119 (69.6) | 68 (71.6) |
| Female | 52 (30.4) | 27 (28.4) |
| Age (years) |  |  |
| < 65 | 101 (59.1) | 56 (58.9) |
| ≥ 65 | 70 (40.9) | 39 (41.1) |
| ECOG performance status score (eCRF source) |  |  |
| 0 | 42 (24.6) | 26 (27.4) |
| 1 | 129 (75.4) | 69 (72.6) |
| Recurrent |  |  |
| No | 106 (62.0) | 53 (55.8) |
| Yes | 65 (38.0) | 42 (44.2) |
| Histological type (Lauren classification) |  |  |
| Intestinal type | 63 (36.8) | 33 (34.7) |
| Diffuse type | 60 (35.1) | 33 (34.7) |
| Others | 7 (4.1) | 6 (6.3) |
| Unknown | 41 (24.0) | 23 (24.2) |
| Number of organs with metastases |  |  |
| < 2 | 41 (24.0) | 19 (20.0) |
| ≥ 2 | 130 (76.0) | 76 (80.0) |
| Number of prior regimens |  |  |
| 2 | 39 (22.8) | 15 (15.8) |
| 3 | 71 (41.5) | 34 (35.8) |
| ≥ 4 | 61 (35.7) | 46 (48.4) |
| PD-L1 expression |  |  |
| ≥ 1% | 7 (4.1) | 7 (7.4) |
| < 1% | 66 (38.6) | 35 (36.8) |
| Missing | 98 (57.3) | 53 (55.8) |
| Diameters of target lesions (mm) |  |  |
| *N* | 136 | 78 |
| Median | 71.0 | 68.0 |
| Time to first progression (months) |  |  |
| Median | 1.45 | 1.41 |
| Mean | 2.93 | 1.82 |
| Poststudy treatment (pharmacotherapy) |  |  |
| Yes | 74 (43.3) | 33 (34.7) |
| Fluoropyrimidine | 24 (14.0) | 13 (13.7) |
| Taxane | 21 (12.3) | 9 (9.5) |
| Platinum | 18 (10.5) | 10 (10.5) |
| Irinotecan | 8 (4.7) | 5 (5.3) |
| Ramucirumab | 19 (11.1) | 4 (4.2) |
| Immunotherapy | 2 (1.2) | 1 (1.1) |
| Other targeted therapies | 4 (2.3) | 4 (4.2) |
| BOR |  |  |
| CR | 0 | 0 |
| PR | 8 (4.7) | 0 |
| SD | 43 (25.1) | 21 (22.1) |
| PD | 76 (44.4) | 55 (57.9) |
| NE | 44 (25.7) | 19 (20.0) |

*BOR* best overall response, *CR* complete response, *ECOG* Eastern Cooperative Oncology Group, *eCRF* electronic case report form, *NE* not evaluated, *PD* progressive disease, *PD-L1* programmed death-ligand 1, *PR* partial response, *SD* stable disease, *TBP* treatment beyond progression

**Online Resource Table 3** Duration of treatment after first PD (months)

|  | **Patients treated beyond progression** | | |
| --- | --- | --- | --- |
|  | **Nivolumab** | **Placebo** | ***P* value** |
| Median (min–max) | 1.12 (0.0–36.5) | 1.08 (0.0–11.1) | 0.52 |
| Mean (SD)  [95% CI] | 2.26 (4.03)  [1.49–3.02] | 1.58 (2.12)  [0.88–2.29] | 0.33 |
| Duration of treatment after first PD (months) by BOR |  | | |
| CR+PR |  |  |  |
| *N* | 14 | - | - |
| Median (min–max) | 4.75 (0.2–10.9) | - | - |
| Mean (SD)  [95% CI] | 4.48 (3.25)  [2.61–6.36] | - | - |
| Stable disease |  |  |  |
| *N* | 27 | 7 |  |
| Median (min–max) | 1.12 (0.0–36.5) | 1.45 (0.0–11.1) | 0.47 |
| Mean (SD)  [95% CI] | 2.73 (6.85)  [0.02–5.44] | 2.99 (3.86)  [0.58–6.55] | 0.92 |
| PD |  |  |  |
| *N* | 48 | 24 |  |
| Median (min–max) | 1.05 (0.0–11.0) | 1.08 (0.0–2.6) | 0.78 |
| Mean (SD)  [95% CI] | 1.67 (2.33)  [0.99–2.35] | 1.06 (0.66)  [0.78–1.34] | 0.21 |

*BOR* best overall response, *CI* confidence interval, *CR* complete response, *max* maximum, *min* minimum, *PD* progressive disease, *PR* partial response, *SD* standard deviation

**Online Resource Table 4** TRAEs of special interest at the 3-year follow-up

|  | **Nivolumab (*n* = 330)** | | **Placebo (*n* = 161)** | |
| --- | --- | --- | --- | --- |
|  | Any grade  *n* (%) | Grade 3 or 4  *n* (%) | Any grade  *n* (%) | Grade 3 or 4  *n* (%) |
| Interstitial lung disease | 6 (1.8) | 1 (0.3) | 0 | 0 |
| Maculopapular rash | 5 (1.5) | 0 | 1 (0.6) | 0 |
| Colitis | 2 (0.6) | 1 (0.3) | 0 | 0 |
| Hyperthyroidism | 2 (0.6) | 0 | 0 | 0 |
| Acute hepatitis | 1 (0.3) | 1 (0.3) | 0 | 0 |
| Autoimmune thyroiditis | 1 (0.3) | 0 | 0 | 0 |
| Hypopituitarism | 1 (0.3) | 1 (0.3) | 0 | 0 |
| Pneumonitis | 2 (0.6) | 1 (0.3) | 0 | 0 |
| Thyroid disorder | 1 (0.3) | 0 | 0 | 0 |

*TRAE* treatment-related adverse event
